# PD‐L1/p‐STAT3 promotes the progression of NSCLC cells by regulating TAM polarization

**DOI:** 10.1111/jcmm.17610

**Published:** 2022-11-13

**Authors:** Rui Zhang, Ziqi Meng, Xuwei Wu, Meihua Zhang, Zhengri Piao, Tiefeng Jin

**Affiliations:** ^1^ Department of Pathology and Cancer Research Center Yanbian University Medical College Yanji China; ^2^ Key Laboratory of the Science and Technology Department of Jilin Province Yanji China; ^3^ Department of Health Examination Centre Yanbian University Hospital Yanji China; ^4^ Department of radiology Yanbian University Hospital Yanji China

**Keywords:** EMT, NSCLC, PD‐L1, STAT3, TAM/M2

## Abstract

PD‐L1 is closely related to the immune escape process of tumour cells, and targeted PD‐L1 clinical immunotherapy has been implemented. However, whether PD‐L1 is involved in TAM/M2 polarization in the TME of NSCLC and its specific mechanism remain unclear. In order to clarify the specific role of PD‐L1 in NSCLC and to seek new treatments for NSCLC, we designed a series of experimental studies. After constructing the co‐culture system and conditioned medium system, the proliferation, apoptosis, metastasis, angiogenesis, EMT process and stemness of NSCLC were detected by MTT, flow cytometry, Transwell, endothelial cell tube formation and western blot assays. The results showed that αPD‐L1 reversed TAM/M2 polarization by suppressing STAT3 phosphorylation in TAM/M2, therapy inhibiting NSCLC cell migration, angiogenesis, EMT process and stemness. However, αPD‐L1 had no effect on the proliferation and apoptosis abilities of NSCLC cells. In vivo experiments showed that αPD‐L1 inhibited lung metastasis of NSCLC and reversed TAM/M2 polarization in TME. The study investigates the mechanism by which PD‐L1 regulates TAMs polarization in TME and promotes malignant progression of NSCLC, providing a new theoretical basis for PD‐L1 targeted therapy of NSCLC.

## 1 INTRODUCTION

Lung cancer is the second most common cancer, and the most common cause of cancer death.^1^ Lung cancer is divided into two groups small cell lung cancer (SCLC) and non‐small cell lung cancer (NSCLC). NSCLC is more common than SCLC, accounting for about 85% of all lung cancer patients.^2^ The clinical treatment of NSCLC includes surgical operation, radiotherapy, chemotherapy, molecular targeting therapy and neoadjuvant therapy.^3^ However, traditional therapeutic methods still have shortcomings, such as large side effects, high recurrence rates and drug tolerance. Thus, the development of innovative approaches such as tumour immunotherapy may provide a new therapeutic strategy for the treatment of NSCLC.

Tumour microenvironment (TME) is the survival condition of tumour cells.^4^ Tumour‐associated macrophages (TAMs) can be polarized into pro‐inflammatory and anti‐cancer M1 type (TAM/M1) or anti‐inflammatory and pro‐cancer M2 type (TAM/M2). TAMs can induce the malignant progression of tumour cells by secreting tumour growth cytokines into TME.^5^ Programmed death ligand 1 (PD‐L1) is the natural ligand of programmed cell death‐1 (PD‐1) and is encoded by the CD274 gene on chromosome 9p24.1. PD‐L1 is mainly expressed in activated T lymphocytes, B lymphocytes, monocytes, mesenchymal stem cell and bone marrow‐derived mast cells etc.^6^ PD‐1/PD‐L1 immune checkpoint or combination with chemotherapy achieved significant efficacy in the treatment of lung cancer.^7^ Studies showed that promoting TAM/M2 polarization may affect the expression of PD‐L1 on NSCLC cells and immune cells, and the invasion behaviour of NSCLC is correlated with the expression of PD‐L1 on tumour immune cells.^8^ Although PD‐L1 and TAM/M2 have been studied extensively, the specific mechanism between PD‐L1 inhibition and TAM/M2 polarization reversal in TME of NSCLC remains unclear.

STAT3, as one of the signal transducers and activators of transcription (STAT) family members, is highly activated in melanoma, breast cancer, lung cancer, pancreatic carcinoma and glioma, suggesting that STAT3 may be an effective target for tumour therapy.^9^ Studies showed that TAMs induce the epithelial–mesenchymal transition (EMT) process through the STAT3 signalling pathway to accelerate the metastasis of colon cancer, revealing the molecular mechanism between immune cells and tumour cells in colon cancer.^10^ STAT3 signalling pathway correlates with the EMT process and stemness in oral squamous cell carcinoma and hepatocellular carcinoma.^11^, ^12^ A Study showed that PD‐L1 reduces sorafenib resistance in hepatocellular carcinoma through STAT3/DNMT1 axis.^13^ In addition, a feedback loop between PD‐L1 and STAT3 is proposed for the first time in this study.

Hence, we designed a series of experiments to investigate the correlation between the progression of NSCLC and the role of PD‐L1 in TAM/M2 polarization, aiming to seek new clinical immunotherapy methods for NSCLC and improve the survival rate of NSCLC patients.

## 2 MATERIALS AND METHODS

### 2.1 Cell lines

Human NSCLC cells (A549, H1299), mouse mononuclear macrophages (RAW264.7), human umbilical vein endothelial cells (HUVECs), human monocyte THP‐1 cell line and mouse Lewis lung cancer (LLC) cells were obtained from Cancer Research Center of Yanbian University in 2020. All cell lines were authenticated by short tandem repeat profiling. All the above cells were cultured using Dulbecco's modified Eagle's medium (DMEM) containing 10% fetal bovine serum (FBS) and 1% penicillin/streptomycin at 37°C, 5% CO_2_. THP‐1 cells were differentiated into macrophages using 100 ng/ml phorbol 12‐myristate 13‐acetate (PMA; Sigma‐Aldrich; Merck KGaA) for 24 h.

### 2.2 Establish co‐culture system

#### 2.2.1 Transwell, ELISA, flow cytometry and western blot assays were performed in the co‐culture system

A549/H1299 and RAW264.7/THP‐1 cells were inoculated in the upper and lower chambers of a Transwell assay, respectively (BD Biosciences). Corresponding drugs were added according to experimental requirements. A549/H1299 cells were used for the Transwell assay, the supernatant of the co‐culture system was harvested for ELISA assay, and RAW264.7/THP‐1 cells were collected for flow cytometry and western blot assays after 48 h.

#### 2.2.2 Wound healing and western blot assays were performed in the co‐culture system

RAW264.7/THP‐1 and A549/H1299 cells were inoculated in the upper and lower chambers of the Transwell assay, respectively. The wound healing assay was carried out when cell growth and fusion reached an appropriate degree. Corresponding drugs were added according to experimental requirements. A549/H1299 cells were collected for western blot assay after 48 h.

### 2.3 Flow cytometry

#### 2.3.1 Macrophage antigen detection

RAW264.7 cells (1 × 10^6^cells/tube) were placed in cell staining buffer solution; 10 μg/ml Fc receptor blocker was added and incubated at 4°C for 10 min. Cell surface fluorescence staining (APC‐CD86, Biolegend) was performed for 20 min at 4°C. Then, RAW264.7 cells were fixed with stationary liquid. Intracellular fluorescence staining (FITC‐CD68, PE‐CD206, Biolegend) was performed after cell membrane perforation. Cells were washed with intracellular staining buffer and centrifuged at 4°C for 5 min. A quantity of 500 μl cell staining buffer solution was added to suspend the cells. Cell analysis was performed by flow cytometer (BD Biosciences).

#### 2.3.2 Apoptosis

The apoptosis kit was manufactured by BD Biosciences. NSCLC cells were treated with conditioned medium for 48 h, and the cells were resuspended by 1 × binding buffer. NSCLC cells were divided into different groups (5 × 10^5^cells/tube), stained, or 55°C pretreatments according to experimental requirements. Annexin V‐FITC and PI (5 μl/tube) was added and incubated for 10 min, respectively. Then, 1 × binding buffer (400 μl/tube) was added and analysed by a flow cytometer.

### 2.4 ELISA assay

The ELISA kit was manufactured by Mlbio. Fifty microlitres of sample and 100 μl of conjugate reagent were added to each well and incubated at 37°C for 60 min. Fifty microliters of developer A and 50 μl of developer B were added to each well at 37°C for 15 min in a dark environment. Fifty microliters of stop solution was added to each well and the absorbance was measured at 450 nm.

### 2.5 Conditioned medium

RAW264.7 cells were treated with PD‐L1 neutralizing antibody (αPD‐L1, 15 mg/ml, Bio X Cell) and/or interleukin‐13 (IL‐13, 30 ng/ml, Biolegend) for 48 h. Cells were cultured in serum‐free medium for 24 h after the supernatant was discarded. Then, the supernatant of RAW264.7 cells was collected and centrifuged for 20 min. The supernatant was collected as the conditioned medium for subsequent experiments. Conditioned medium was classified into control, IL‐13 and IL‐13 + αPD‐L1 groups according to different treatments.

### 2.6 MTT assay

NSCLC cells (5 × 10^3^cells/well) were inoculated in 96‐well plates treated with different conditioned media for 0, 24, 48 and 72 h, respectively. Twenty microliters of MTT (5 mg/ml) and 80 μl of DMEM were added to each well at 37°C and 5% CO_2_ for 4 h and then dimethylsulphoxide was added to each well. The absorbance was measured at 490 nm.

### 2.7 Wound healing assay

NSCLC cells were inoculated in six‐well plates. A 200‐μl pipette tip was used for scratch treatment when the cells grew and fused to an appropriate degree. According to the experimental requirements, the corresponding conditioned medium or drug was added to the well. The degree of wound healing was recorded using an inverted microscope (Olympus) at 0, 12 and 24 h. Image J software (NIH) was used to measure and analyse the wound widths.

### 2.8 Transwell assay

NSCLC cells (5 × 10^4^cells/well) were inoculated in the upper chambers of the Transwell assay, and conditioned medium or RAW264.7/THP‐1 cells was added into the lower chambers and cultured at 37°C for 12 h. Paraformaldehyde was fixed, haematoxylin was stained and the unmigrated cells were erased. Five visual field images were randomly selected under the microscope (200×) for statistical analysis.

### 2.9 Endothelial cell tube formation assay

Fifty microliters of Matrigel (BD Biosciences) mixture was added to a 96‐well plate (serum‐free medium: Matrigel = 1: 1) and placed in incubator until Matrigel mixture has completely solidified. HUVECs (1.5 × 10^4^cells/well) were inoculated in a 96‐well plate and cultured at 37°C. An inverted microscope was used to observe and capture images of microvascular formation at 6 h.

### 2.10 Western blot assay

Appropriate RIPA lysate containing protease and phosphatase inhibitor cocktails (CWBio Biosciences) were added to lysate the cells. Standard curves were drawn with BSA for protein quantitation. Then, protein denaturation was performed. After isolation in SDS–PAGE, the denatured protein was transferred into the immobilon‐polyvinylidene fluoride (PVDF) membrane (Millipore). PVDF membranes were incubated with the corresponding primary antibody at 4°C overnight. Primary antibody includes β‐actin (CWBio Biosciences), CD31, vascular endothelial growth factor (VEGF), matrix metalloproteinase 9 (MMP9), matrix metalloproteinase 2 (MMP2), ZO‐1, E‐cadherin, vimentin, Snail, Slug, Twist, CD44, Oct4, Sox2, Bmi‐1, Nanog, p‐STAT3, STAT3, p‐STAT6, STAT6 (Santa Cruz Biotechnology). Then, membranes were incubated with secondary antibody at RT for 1 h. The gel imaging system (Bio‐Rad) was used to collect the image.

### 2.11 Immunofluorescence

NSCLC cells were inoculated in a six‐well plate containing cover glass. Cells were fixed with 4% paraformaldehyde, permeabilized with 0.5% TritonX‐100 (CWBio Biosciences), blocked with 3% BSA (Solarbio), and incubated with primary antibodies overnight at 4°C. Then, cells were incubated with the corresponding secondary antibody for 2 h. Seal the cells with anti‐fade fluorescence mounting medium containing 4′,6‐diamidino‐2‐phenylindol (DAPI, Solarbio), acquisition of fluorescent signals by microscopy.

### 2.12 Animal model

A total of 20 C57BL/6 mice (4–6 weeks old) used in this study were purchased from Beijing Vital River Laboratory Animal Technology Co., Ltd. Ten C57BL/6 mice, and LLC cells (5 × 10^6^cells) were used to construct a subcutaneous xenograft model by subcutaneous injection. The xenograft mice were randomly divided into two groups (control group *n* = 5, αPD‐L1 group *n* = 5). The xenograft mice of the control group were treated with PBS and the αPD‐L1 group was treated with αPD‐L1 (200 μg) by tail intravenous injection every 3 days for seven times.^14^ Tumour size was measured and the tumour volume was calculated according to the formula (length×width^2^ × 0.5). The mice were sacrificed 28 days after PBS or αPD‐L1 treatment. Tumour tissue and lung tissue of mice were obtained. Ten C57BL/6 mice and LLC cells (1 × 10^6^cells) were used to construct a lung metastasis model by tail intravenous injection. The mice were randomly divided into two groups (control group *n* = 5, αPD‐L1 group *n* = 5) and treated with PBS or αPD‐L1 (200 μg), respectively. The mice were sacrificed 28 days after PBS or αPD‐L1 treatment. Lung tissue of mice was obtained. The tissues were fixed with 10% formalin and embedded in paraffin for further haematoxylin and eosin (HE) staining or immunohistochemical analysis. All experiments were approved by the Animal Ethics Committee of Yanbian University.

### 2.13 Haematoxylin and eosin (HE) staining

Tissue sections were stained with haematoxylin solution for 5 min after deparaffinization and rehydration, and then the tissue sections were stained with eosin solution for 3 min. Dehydrated through increased concentrations of ethanol and xylene. Capture images by microscope.

### 2.14 Immunohistochemical (IHC) analysis

Tissue sections were dewaxed and rehydrated. After antigen retrieval by sodium citrate buffer, it was incubated with 3% hydrogen peroxide (H_2_O_2_) (ZSGB‐BIO) for 30 min. Then, tissue sections were incubated with corresponding primary antibodies (CD206, E‐Cadherin, Vimentin, VEGF, CD44) overnight at 4°C. The next day, tissue sections were incubated with secondary antibodies. The tissues were successively stained with 3,3′‐diaminobenzidine (DAB) (ZSGB‐BIO) and haematoxylin solution.

### 2.15 Statistical analysis

GraphPad Prism 8.0 software (GraphPad) was used for all statistical analyses. The experimental results were averaged and expressed as mean ± standard deviation. The mean of two samples or multiple group samples was compared by Student's *t*‐test and one‐way anova, independently. *p*‐Value < 0.05 was statistically significant (*p < 0.05, **p < 0.01, ***p < 0.001, or ****p < 0.0001). *p*‐Value > 0.05 has no significance (ns). All experiments were repeated three times.

## 3 RESULTS

### 3.1 αPD‐L1 reverses NSCLC cells‐induced TAM/M2 polarization

**FIGURE 1:**
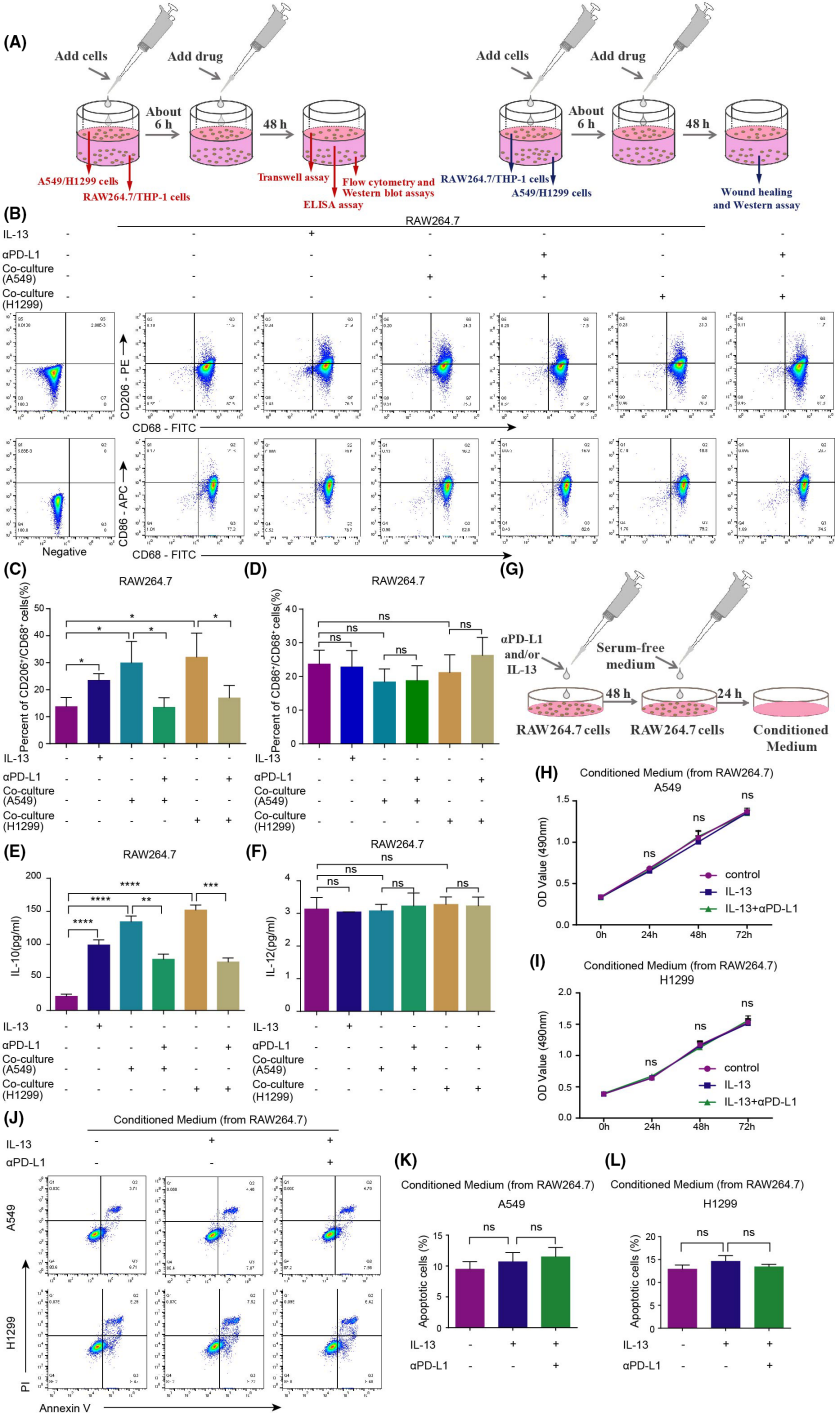
Efficiency of αPD‐L1 on TAMs polarization. (A) The pattern diagram of the co‐culture system establishment; (B–D) Efficiency of A549/H1299 cells and αPD‐L1 on TAMs polarization was detected by the flow cytometry assay; (E) The secretion level of IL‐10 was detected in the RAW264.7 co‐culture system by the ELISA assay; (F) The secretion level of IL‐12 was detected in the RAW264.7 co‐culture system by the ELISA assay; (G) The pattern diagram of conditioned medium; (H–I) The proliferation ability of NSCLC cells was detected by the MTT assay; (J–L) The apoptosis ability of NSCLC cells was detected by the flow cytometry assay.

The co‐culture system of NSCLC and RAW264.7 cells were established, and the effect of αPD‐L1 on the polarization of RAW264.7 cells was detected in this system (Figure 1A). The expression of TAM/M2 marker CD206 in RAW264.7 cells was detected by flow cytometry. The results showed that the expression of CD206 was significantly up‐regulated in the co‐culture group, while the expression of CD206 was down‐regulated after adding αPD‐L1 into the co‐culture system. αPD‐L1 had no effect on the expression of TAM/M1 marker CD86 (Figure 1B–D).

To verify the effect of αPD‐L1 on the TAM/M2 polarization, the concentration of related cytokines in the co‐culture system was detected by ELISA assay. The results showed that IL‐10 (TAM/M2 secretion) was significantly increased, while the secretion of IL‐10 was decreased after αPD‐L1 into the co‐culture system (Figure 1E). However, αPD‐L1 had no effect on IL‐12 (TAM/M1 secretion) levels in the co‐culture system (Figure 1F). These results suggested that αPD‐L1 reverses NSCLC cells‐induced TAM/M2 polarization, but had no effect on TAM/M1 polarization.

### 3.2 αPD‐L1 has no effect on the proliferation and apoptosis ability of NSCLC

A549 or H1299 cells were treated with conditioned medium. The conditioned medium model is shown in Figure 1G. The proliferation ability of NSCLC cells was detected by the MTT assay (Figure 1H,I). The result showed that the proliferation ability was not significantly changed. Then, apoptosis was detected by the flow cytometry assay (Figure 1J–L). There was no change in apoptosis ability of A549 and H1299 cells either between the control group and the IL‐13 group, or between the IL‐13 group and IL‐13 + αPD‐L1 group. Results suggested that conditioned medium produced by αPD‐L1 and/or IL‐13 had no effect on the proliferation and apoptosis ability of NSCLC cells.

### 3.3 αPD‐L1 inhibits migration and angiogenesis of NSCLC by reversing TAM/M2 polarization

**FIGURE 2:**
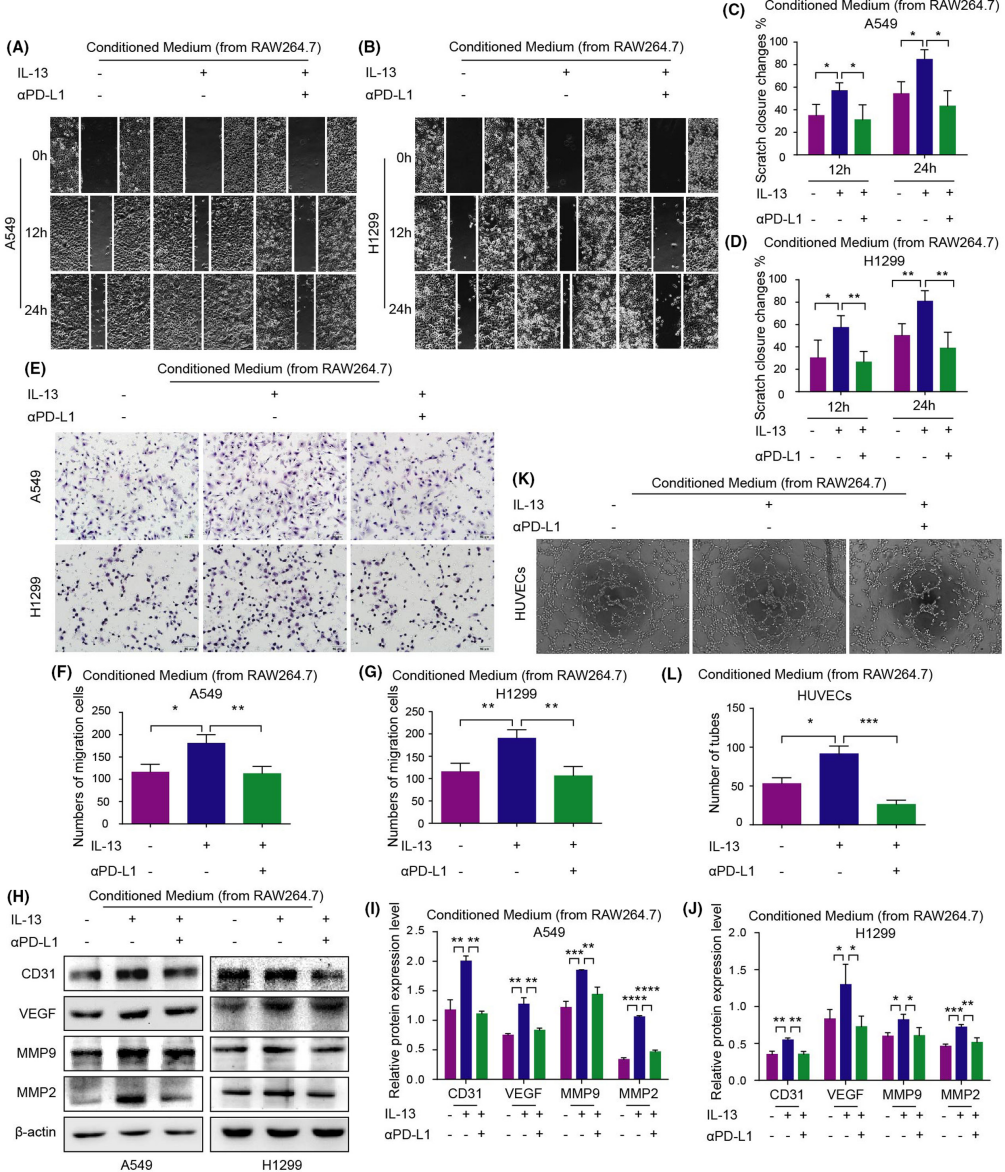
Efficiency of migration and angiogenesis ability of NSCLC after conditioned medium treatment. (A–D) The wound healing ability of NSCLC cells treated with IL‐13 and αPD‐L1 were detected by the wound healing assay. (E–G): The migration ability of NSCLC cells treated with IL‐13 and αPD‐L1 were detected by the Transwell assay. (H–J) Expression levels of related proteins in NSCLC cells were detected by the western blot assay. (K–L) The microvascular formation of HUVECs treated with conditioned medium was detected by the endothelial cell tube formation assay.

Migration is closely associated with poor prognosis.^15^ Therefore, the migration ability of NSCLC cells treated with conditioned medium was detected using the wound healing and transwell assays. The result showed that the A549 or H1299 cells migration ability of the IL‐13 group was promoted compared with the control group. However, αPD‐L1 could inhibit the IL‐13‐induced migration ability of A549 and H1299 cells (Figure 2A–G). Similarly, αPD‐L1 inhibited the protein expression of metastasis‐related markers MMP9 and MMP2 in NSCLC cells by reversing TAM/M2 polarization (Figure 2H–J).

Angiogenesis plays an important role in cancer metastasis.^16^ Hence, HUVECs tube formation ability and the protein expression of vascular markers (CD31, VEGF) after being treated with different conditioned mediums were detected by endothelial cell tube formation and western blot assay, respectively (Figure 2H–L). Compared with the control group, the angiogenesis ability of the IL‐13 group was promoted, while this ability of the IL‐13 + αPD‐L1 group was inhibited.

**FIGURE 3:**
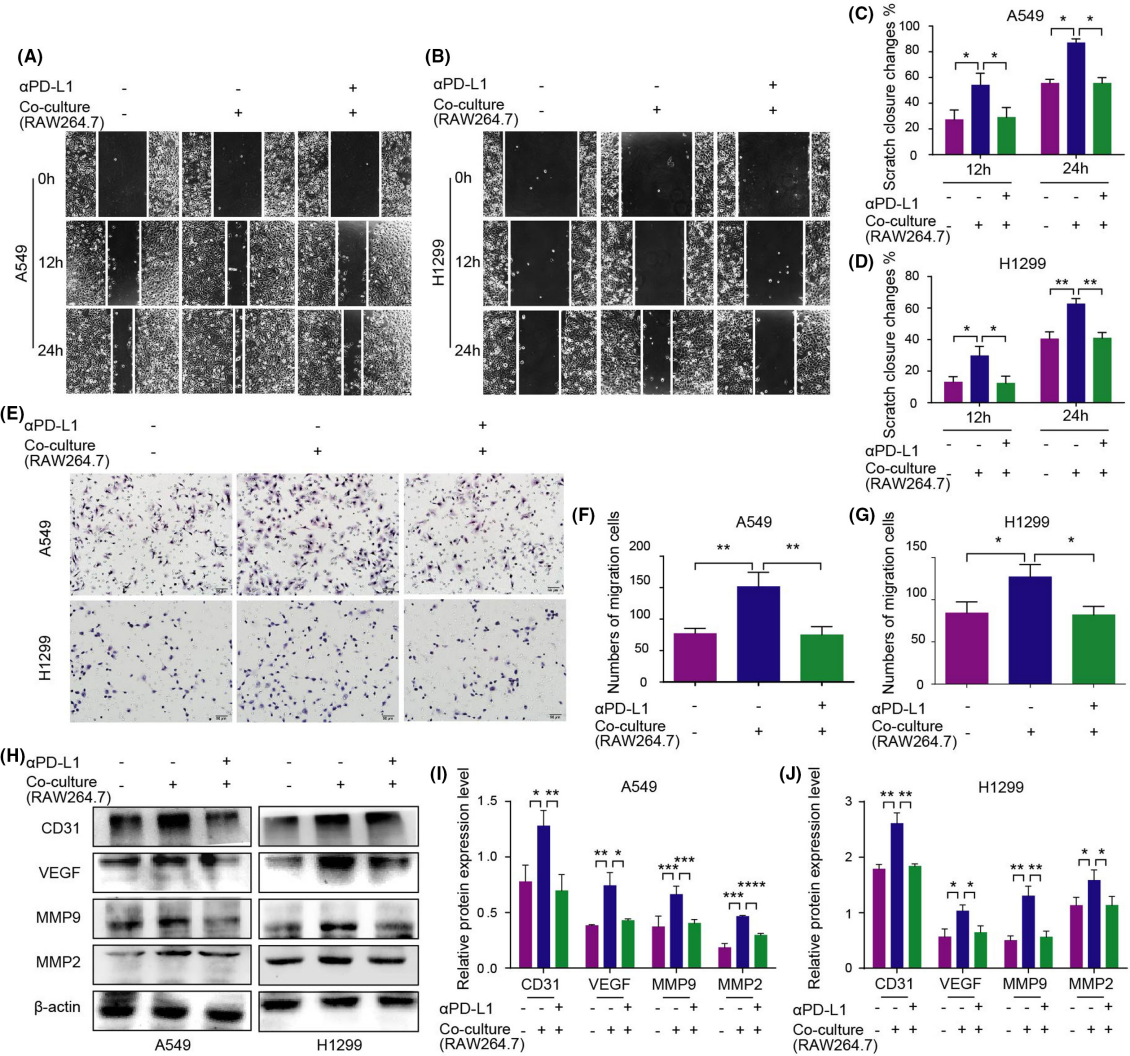
Efficiency of TAM/M2 polarization and αPD‐L1 on NSCLC migration and angiogenesis in the RAW264.7 co‐culture system. (A–D) The wound healing ability of NSCLC cells treated with TAMs or αPD‐L1 in the RAW264.7 co‐culture system was detected by the wound healing assay. (E–G) The migration ability of NSCLC cells treated with TAMs or αPD‐L1 in the RAW264.7 co‐culture system was detected by the Transwell assay. (H–J) Expression levels of migration and angiogenesis‐related markers in the RAW264.7 co‐culture system were detected by the western blot assay.

A similar study was conducted to investigate whether the migration and angiogenesis of NSCLC were altered in the co‐culture system. The results showed that TAM/M2 co‐culture system increased the migration rate and angiogenesis of NSCLC cells, while αPD‐L1 treatment inhibited this phenomenon (Figure 3A–J; Figure S1A‐J).

### 3.4 αPD‐L1 inhibits the EMT process of NSCLC by reversing TAM/M2 polarization

**FIGURE 4:**
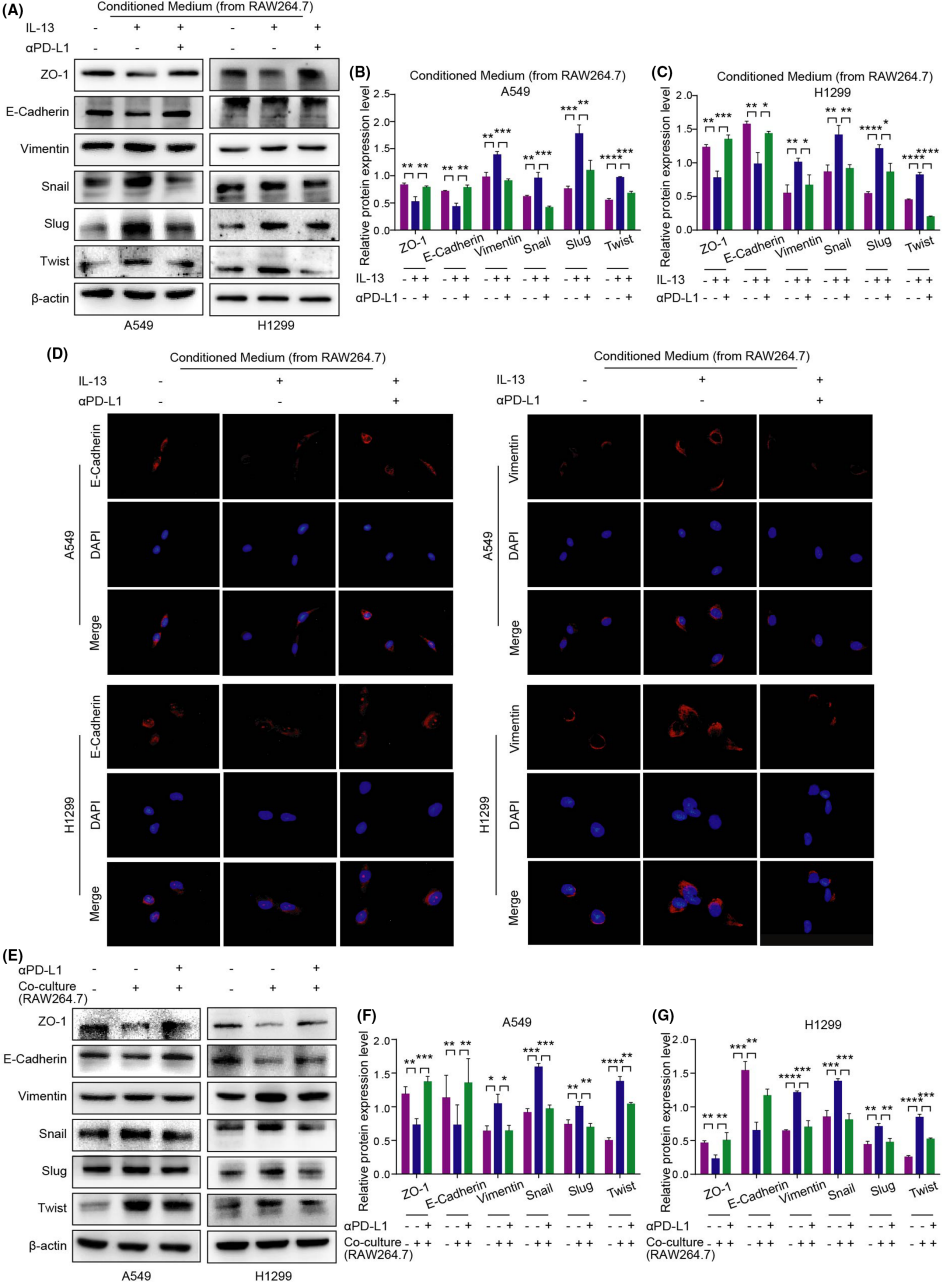
Correlation between TAM/M2 polarization and EMT process in NSCLC cells. (A–C) Expression of EMT‐related markers treated with conditioned medium were detected by the western blot assay. (D) The fluorescence signal intensity of E‐cadherin (expressed in the cytoplasm) and vimentin (expressed in the cytoplasm) treated with conditioned medium in A549/H1299 cells was detected by Immunofluorescence. (E–G) Expression of EMT markers of A549/H1299 cells in the RAW264.7 co‐culture system were detected by the western blot assay.

To investigate the effect of TAM/M2 and αPD‐L1 on the EMT process of NSCLC, the protein expression level of EMT‐related markers in A549 and H1299 cells were detected and treated with conditioned medium by the western blot assay (Figure 4A–C). The results showed that the protein expression levels of epithelial markers ZO‐1 and E‐cadherin in the IL‐13 group were down‐regulated compared with the control group, while the expression levels of mesenchymal markers vimentin, Snail, Slug and Twist were up‐regulated. Contrasted with the IL‐13 group, the protein expression of epithelial markers was enhanced in the IL‐13 + αPD‐L1 group, and the expression of mesenchymal markers was reduced. The results were further confirmed by immunofluorescence assay (Figure 4D). We found that IL‐13 reduced the fluorescence signal of E‐Cadherin and enhanced the fluorescence signal of vimentin. However, the expression of fluorescence signal in the IL‐13 + αPD‐L1 group was the opposite.

EMT‐related markers of A549 and H1299 cells were detected in the co‐culture system by the western blot assay (Figure 4E–G; Figure S1H‐J). The results showed that TAM/M2 polarization down‐regulated the protein expression levels of epithelial markers and up‐regulated the protein expression levels of mesenchymal markers, while this phenomenon was reversed after αPD‐L1 treatment.

### 3.5 αPD‐L1 inhibits the stemness of NSCLC cells by reversing TAM/M2 polarization

**FIGURE 5:**
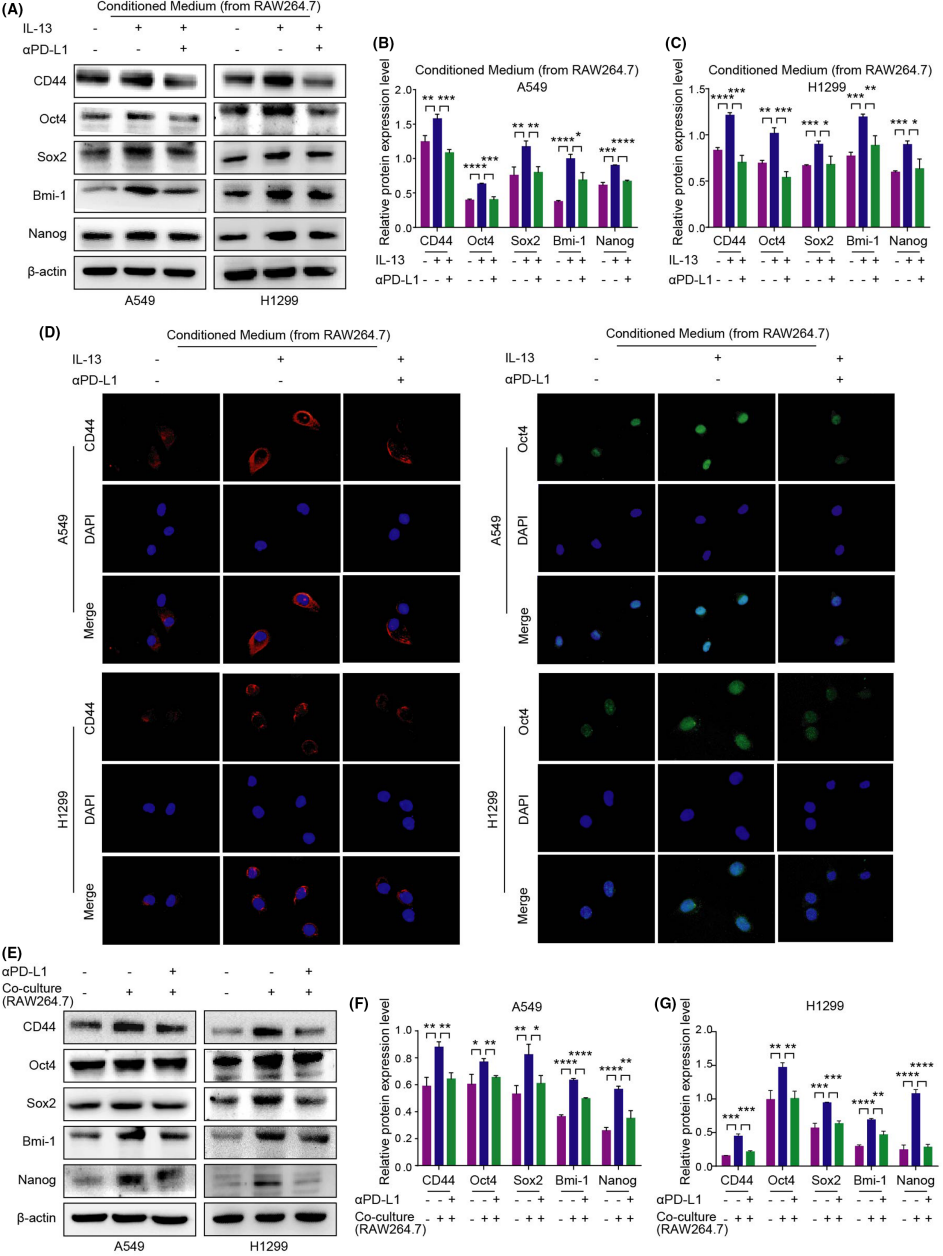
Correlation between TAM/M2 polarization and stemness markers in NSCLC cells. (A–C) Expression of stemness‐related markers treated with conditioned medium were detected by the western blot assay. (D) The fluorescence signal intensity of CD44 (expressed in the cytoplasm) and Oct4 (expressed in the nucleus) in NSCLC cells were detected by the immunofluorescence assay. (E–G) Expression of stemness markers of A549/H1299 cells in the RAW264.7 co‐culture system were detected by the western blot assay.

Cancer stemness is closely related to the occurrence and metastasis of NSCLC.^17^ To investigate the effect of αPD‐L1 on stemness of NSCLC cells by regulating TAM/M2 polarization, the protein expression of stemness‐related markers, including CD44, Oct4, Sox2, Bmi‐1 and Nanog were detected by the western blot assay (Figure 5A–C,E–G; Figure S1K‐M). The results showed that the protein expression of stemness‐related markers was up‐regulated after TAM/M2 polarization induced by IL‐13 or NSCLC cells, while αPD‐L1 inhibited this phenomenon. The fluorescence signal intensity was detected by immunofluorescence assay (Figure 5D). The result showed that the fluorescence signal intensity of CD44 and Oct4 was up‐regulated after TAM/M2 polarization, while αPD‐L1 down‐regulated the fluorescence signal intensity of CD44 and Oct4.

### 3.6 STAT3 participates in PD‐L1‐induced TAM/M2 polarization

**FIGURE 6:**
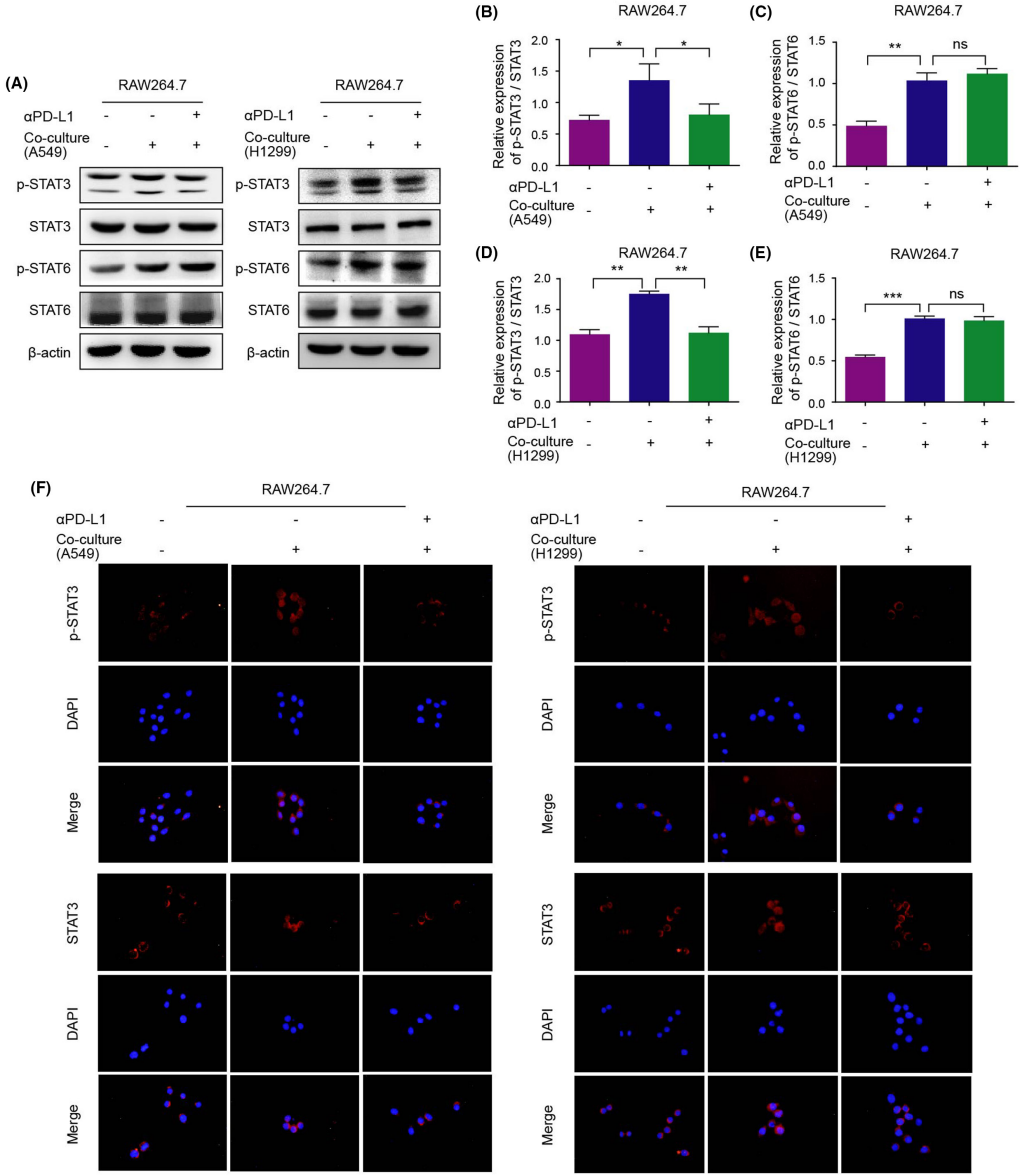
Specific mechanism of TAM/M2 polarization in the co‐culture system. (A–E): Expression levels of p‐STAT3/STAT3 and p‐STAT6/STAT6 in RAW264.7 cells in the co‐culture system were detected by the western blot assay. (F) The fluorescence signal intensity of p‐STAT3 (expressed in cytoplasm and nucleus) and STAT3 (expressed in cytoplasm and nucleus) of RAW264.7 cells in the co‐culture system were detected by the immunofluorescence assay.

The above findings indicated that αPD‐L1 inhibits the progression of NSCLC by regulating TAM/M2 polarization. To explore the molecular mechanism of αPD‐L1 inhibiting TAM/M2 polarization induced by NSCLC cells, we detected the changes in signalling pathway protein expression of RAW264.7 cells in the co‐culture system (Figure 6A–E). The results showed that the protein expression levels of p‐STAT3 and p‐STAT6 of the co‐culture group were increased in RAW264.7 cells, while the protein expression levels of STAT3 and STAT6 were not affected. Compared with the A549 or H1299 cells co‐culture group, the protein expression level of p‐STAT6 in RAW264.7 cells had no significant change after αPD‐L1 treatment in the co‐culture system, while the protein expression level of p‐STAT3 was reduced. We detected the changes of STAT3 signalling pathway protein expression of THP‐1 cells in the co‐culture system, and similar results were obtained (Figure S2A‐C).

Subsequently, we detected the fluorescence signal intensity of STAT3 and p‐STAT3 by the immunofluorescence assay (Figure 6F). After the co‐culture of RAW264.7 with A549 or H1299 cells, the fluorescence signal intensity of p‐STAT3 was enhanced and is mainly expressed in the nucleus. This phenomenon was reversed after αPD‐L1 treatment in the co‐culture system. Although STAT3 fluorescence signal intensity was not affected by NSCLC cells or αPD‐L1, STAT3 was expressed in the nucleus after co‐culture with NSCLC cells.

### 3.7 αPD‐L1 inhibits NSCLC metastasis by reversing TAM/M2 polarization

**FIGURE 7:**
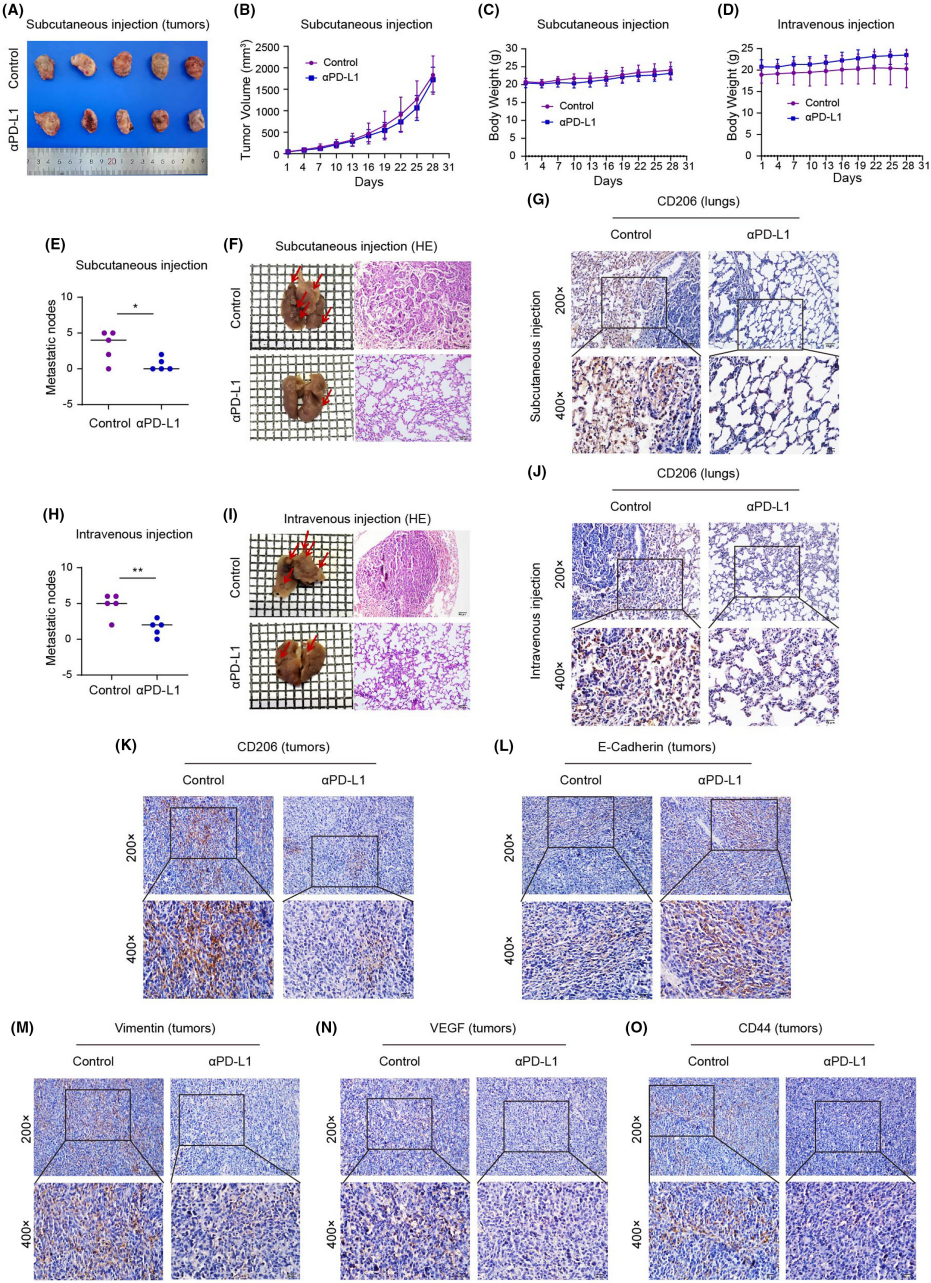
Effect of αPD‐L1 on NSCLC lung metastasis and TAM/M2 polarization in vivo. (A–B) Tumour size and volume of subcutaneous injection; (C–D) Body weight of mice by subcutaneous injection and intravenous injection; (E–F): the number of lung metastatic nodes and haematoxylin and eosin (HE) staining of lung tissues after subcutaneous injection; (G) Expression of CD206 in lung tissues after subcutaneous injection detected by IHC; (H–I) The number of lung metastatic nodes and HE staining of lung tissues after intravenous injection; (J) Expression of CD206 in lung tissues after intravenous injection detected by immunohistochemical (IHC) analysis; (K–O) Expression of CD206, E‐cadherin, vimentin, VEGF and CD44 in tumour tissues after subcutaneous injection detected by IHC

The subcutaneous xenograft model and lung metastasis model have constructed for evaluating the effect of αPD‐L1 on TAM/M2 polarization and NSCLC metastasis in vivo. Results showed that αPD‐L1 had no effect on tumour size, tumour volume and body weight (Figure 7A–D). However, αPD‐L1 can reduce the number of metastatic nodes on lung tissue in the subcutaneous xenograft model and lung metastasis model. HE staining also confirmed that αPD‐L1 can inhibit lung metastasis of NSCLC (Figure 7E,F,H,I). The result of immunohistochemical (IHC) analysis showed that αPD‐L1 can weaken the expression of CD206 in lung tissues and tumour tissues (Figure 7G,J,K). This indicates that the role of αPD‐L1 in reverses TAM/M2 polarization in TME in vivo. αPD‐L1 enhances the protein expression of E‐cadherin, and weakens the protein expression of vimentin, VEGF and CD44 in tumour tissues (Figure 7L–O).

## 4 DISCUSSION

Immunotherapy as a new treatment method has the advantages of strong targeted and small side effects compared with chemotherapy or traditional therapy.^18^ Immunotherapy inhibits the malignant progression of tumours by reshaping TME.^19^ A study showed that TAMs were the main reason for maintaining the expression of PD‐L1 in TME after the disappearance of PD‐L1 on tumour cells.^20^ And PD‐L1 has expression on TAMs of NSCLC. The number of NSCLC patients that PD‐L1 positive on TAMs is slightly greater than PD‐L1 negative on TAMs.^21^ Therefore, the expression of PD‐L1 on TAMs can be used as a new target for cancer immunotherapy. Studies showed that TME regulates the sensitivity of lung cancer to enhance PD‐1/PD‐L1 blocking therapy, suggesting that the response of cancer cells to immunotherapy is closely related to TME.^22^ However, the effect of PD‐L1 on TAM/M2 polarization in TME of NSCLC has not been reported. IL‐13 can significantly induce TAM/M2 polarization so we use it as a positive control in this study.^23^ We found that NSCLC cells induce TAM/M2 polarization, while αPD‐L1 can reverse this phenomenon in vitro and in vivo. There is no effect on TAM/M1 polarization.

Metastasis is the main cause of cancer‐related death.^24^ A study showed that TAMs can secrete exosomal to promote metastasis of NSCLC.^25^ Anti‐PD‐L1 therapy has been widely used in the first‐line treatment of advanced lung cancer metastasis patients.^26^ Tumour angiogenesis plays an important role in tumour metastasis.^27^ PD‐L1 inhibitors significantly inhibit angiogenesis in embryos.^28^ TAM/M2 polarizes to TAM/M1 can inhibit VEGF expression and angiogenesis, and inhibition of angiogenesis can reshape TME so that promote the polarization of TAM/M2 to TAM/M1.^29^ However, the role of PD‐L1 in TAM/M2 polarization‐induced malignant progression of NSCLC remains unclear. In this study, we found that TAM/M2 polarization promoted the migration and angiogenesis of NSCLC, while αPD‐L1 inhibited the malignant progress of NSCLC in vitro. αPD‐L1 and/or TAM/M2 polarization had no effect on the proliferation and apoptosis of NSCLC cells in vitro. αPD‐L1 inhibited the lung metastasis of NSCLC in vivo.

Epithelial–mesenchymal transition is closely related to biological processes such as tumour genesis, metastasis and angiogenesis.^30^ PD‐L1 is associated with tumour metastasis and the EMT process.^31^ The EMT process of colon cancer can be inhibited by blocking crosstalk between colon cancer cells and TAMs.^32^ We found that TAM/M2 polarization promoted the EMT process of NSCLC cells. However, reversal of TAM/M2 polarization by αPD‐L1 can inhibit the EMT process of NSCLC, thus inhibiting the malignant progression of NSCLC such as metastasis and vascular formation in vitro and in vivo.

Stemness is a crucial biological process that can drive tumour cell metastasis.^33^ A study showed that the invasion, EMT process, and spheres formation of colon cancer cells is enhanced but have little effect on the proliferation of colon cancer spheroid cells in vitro.^34^ Prostate cancer stem cells are involved in cancer metastasis rather than proliferation.^35^ Our data showed that TAM/M2 polarization enhanced the stemness of NSCLC cells, while αPD‐L1 can reduce the stemness of NSCLC cells by reversing TAM/M2 polarization in vitro and in vivo.

Then, we investigate the specific molecular mechanism by which αPD‐L1 reverses TAM/M2 polarization. A study found that gefitinib inhibits TAM/M2 polarization in Lewis lung cancer by targeting the STAT6 signalling pathway.^36^ Dioscin inhibits TAM/M2 polarization through JNK and STAT3 signalling pathways, thereby inducing anti‐tumour immunity and inhibiting lung cancer angiogenesis.^37^ In this study, we found that αPD‐L1 reverses TAM/M2 polarization by inhibiting STAT3 phosphorylation. The key activation mechanism of STAT3 transcriptional function is two phosphorylated STAT3 monomers, which form homodimer through the interaction of the SH2 domain, then enters the nucleus for regulating biological function.^38^ Hence, we detected the localization of STAT3 and found that p‐STAT3 entry into the nucleus. TAMs promote the malignant progression of cancer by producing cytokines such as IL‐10 in TME.^23^ And IL‐10 can promote STAT3 activation.^39^ Our study showed that αPD‐L1 can inhibit IL‐10 secretion of TAM/M2. Therefore, αPD‐L1 possibly suppress STAT3 activation and malignant progression of NSCLC by inhibiting IL‐10 secretion of TAM/M2.

## 5 CONCLUSIONS

In summary, we discussed that αPD‐L1 blocks the signal crosstalk between NSCLC cells and TAMs by inhibiting STAT3 phosphorylation of TAM/M2 in vitro and in vivo, thus slowing down the EMT process, stemness, migration, angiogenesis and lung metastasis of NSCLC cells in vitro. The results of this study might provide a new therapeutic strategy for the clinical diagnosis and treatment of NSCLC.

## AUTHOR CONTRIBUTIONS

**Rui Zhang:** Formal analysis (equal); software (equal); visualization (equal); writing – original draft (equal). **Ziqi Meng:** Methodology (equal); validation (equal); visualization (equal); writing – original draft (equal). **Xuwei Wu:** Methodology (equal); software (equal); writing – original draft (equal). **Meihua Zhang:** Funding acquisition (equal); investigation (equal); resources (equal); supervision (equal); writing – review and editing (equal). **Zhengri Piao:** Funding acquisition (equal); project administration (equal); resources (equal); writing – review and editing (equal). **Tiefeng Jin:** Conceptualization (equal); data curation (equal); investigation (equal); project administration (equal); supervision (equal); writing – review and editing (equal).

## FUNDING INFORMATION

This study was supported by grants from the National Natural Science Foundation of China (No. 81960554, No.82060554).

## CONFLICT OF INTEREST

The authors declare no conflicts of interest.

## Supporting information


FigureS1

FigureS2

## Data Availability

Data Availability; The datasets used and analyzed during the current study are available from the corresponding author upon reasonable request.
