# Supplementary figures and images for: PD‐L1/p‐STAT3 promotes the progression of NSCLC cells by regulating TAM polarization
Source: J Cell Mol Med. 2022 Nov 13;26(23):5872–86. doi: 10.1111/jcmm.17610 (PMC9716221; doi:10.1111/jcmm.17610)

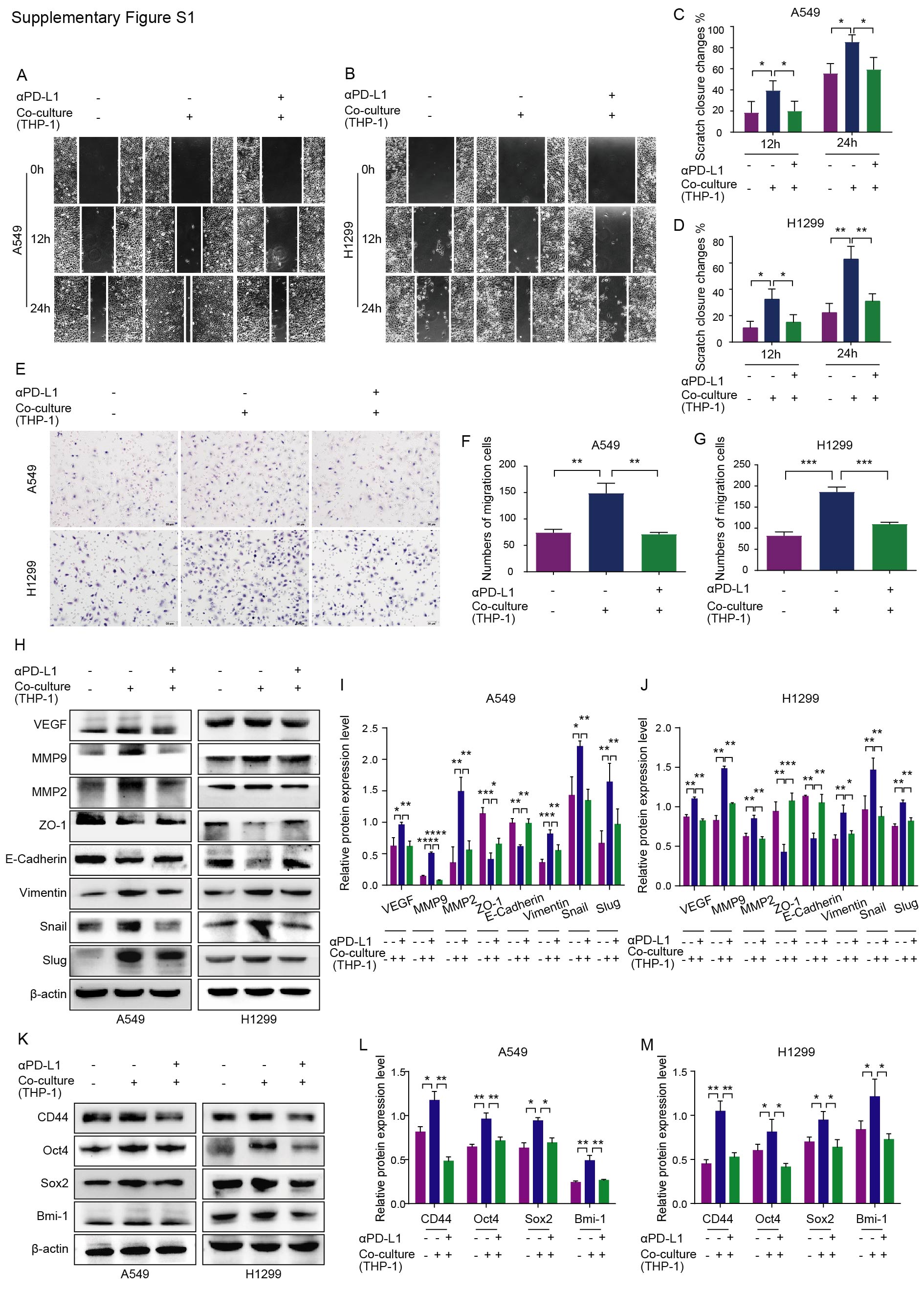

Supplement: Supplementary file 1 — FigureS1 [file JCMM-26-5872-s001.jpg]

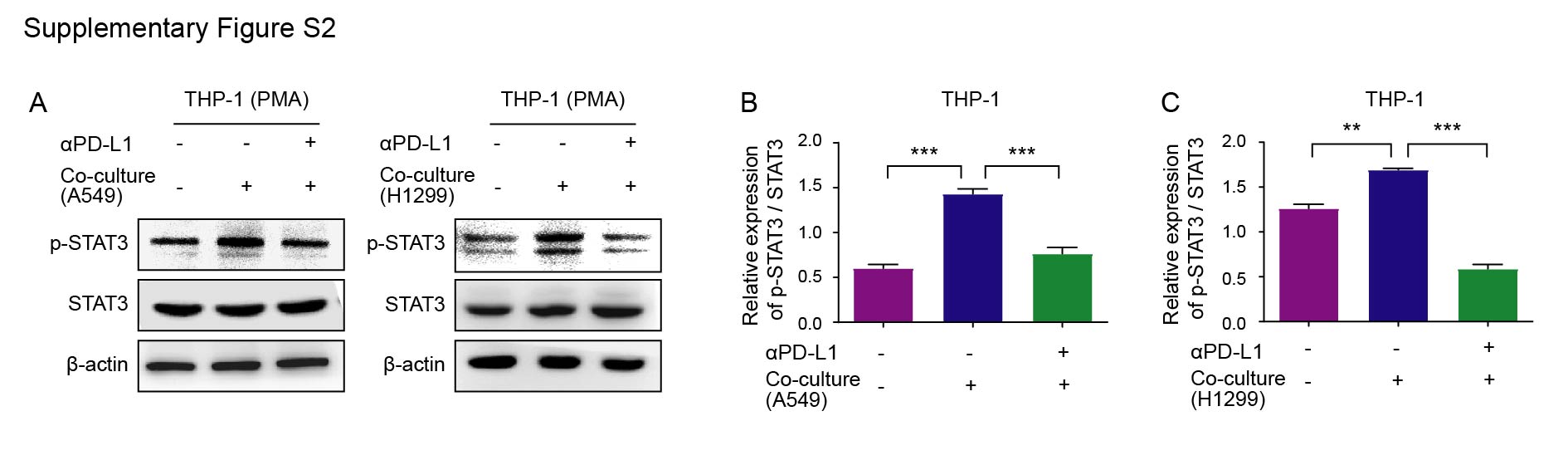

Supplement: Supplementary file 2 — FigureS2 [file JCMM-26-5872-s002.jpg]
